# Regulation of mitochondrion-associated cytosolic ribosomes by mammalian mitochondrial ribonuclease T2 (RNASET2)

**DOI:** 10.1074/jbc.RA118.005433

**Published:** 2018-11-01

**Authors:** Jinliang Huang, Peipei Liu, Geng Wang

**Affiliations:** From the Ministry of Education Key Laboratory of Bioinformatics, Cell Biology and Development Center, School of Life Sciences, Tsinghua University, Beijing 100084, China

**Keywords:** mitochondria, mitochondrial metabolism, RNA turnover, RNA transport, enzyme, cytosolic ribosome, mitochondrial RNA import, mtRNA degradation, ribosomal RNA degradation, synchronized translation

## Abstract

Mitochondrial proteins are encoded in both mitochondrial and nuclear genomes. The expression levels of these two pools of mitochondrial genes are co-regulated and synchronized. Import and assembly of the nucleus-encoded oxidative phosphorylation (OXPHOS) subunits affect protein synthesis in the mitochondrial matrix by engaging the mitochondrial ribosomes. How the ribosomes at the outside of mitochondria are regulated by mitochondria, however, remains mostly unexplored. Here, using an array of biochemical assays and genetic knockdown and overexpression in HEK293 or mouse cells, we show that cytosolic rRNAs that are associated with the mitochondrial outer membrane have very different decay patterns from those of both endoplasmic reticulum–associated and –nonassociated cytosolic rRNAs. Mitochondrial intermembrane space RNase T2 (RNASET2), which has been previously shown to degrade mitochondrial RNAs, is also responsible for selective degradation of the cytosolic rRNAs on the outer membrane. We noted that the degradation activity also has a positive effect on nuclear transcription of rRNAs, suggesting a compensatory feedback mechanism, and affects protein translations in and out of mitochondria. These findings establish a mechanism for the co-regulation of gene expression programs inside and outside of mitochondria in mammalian cells.

## Introduction

Mitochondria are the main source of cellular energy and also play essential roles in cellular processes such as apoptosis (1). Mitochondrial energy output and functions are constantly regulated to meet the demands of intracellular and extracellular changes. The integrity of mitochondria is also constantly challenged by protein damage, nucleic acid damage, and lipid damage. A fine balance of biosynthesis, repair, and recycling is needed to maintain mitochondrial functions.

The majority of mitochondrial macromolecules are encoded in the nuclear genome, synthesized outside of mitochondria, imported into mitochondria, and assembled or processed into functional products (2–4). Mitochondria, however, still maintain a small genome. In mammals, this genome encodes 2 rRNAs, 22 tRNAs, and 13 core subunits of the OXPHOS^2^ complexes (5). Transcription of the mitochondrial genome is performed by a single-subunit RNA polymerase (6). Transcripts are polycistronic, and the mRNAs are translated by a protein-rich mitoribosome (7, 8). In mammals, mitochondrial RNAs (mtRNAs) are degraded by a complex RNA degradation machinery with components including SUV3p, PDE12, and LACTB2 in the matrix; REXO2 and PNPASE in both the matrix and the mitochondrial intermembrane space (IMS); and RNASET2 in the IMS (2, 9–13). Because the two mitochondrial rRNAs are constantly degraded, the RNA degradation machinery not only regulates mitochondrial mRNA levels but also plays a role in regulating the mitoribosome functions (9). Even though nucleus-encoded mitochondrial genes and mitochondrion-encoded genes are expressed by distinct machineries and regulated by different mechanisms, these two gene expression programs are synchronized through a yet-unclear mechanism (14).

Localized translation of proteins has been shown to coordinate protein synthesis with cellular functions (15–17). Both nucleus-encoded mRNAs and cytosolic ribosomes have been shown to be associated with mitochondrial outer membrane (18–20). Localized translation coupled with mitochondrial protein import has been suggested to contribute to complex assembly within mitochondria (21–23). Recent discoveries have shown that import and assembly of nucleus-encoded OXPHOS subunits regulate mitochondrial protein synthesis in the matrix, hence providing a mechanism for synchronizing mitochondrial translation to the influx of nucleus-encoded proteins (24–26). Whether mitochondria could regulate the cytosolic ribosomes on the mitochondrial outer membrane, however, remains unexplored. Here we demonstrate that mitochondrion-associated cytosolic rRNAs have very different decay patterns from both endoplasmic reticulum–associated and –nonassociated cytosolic rRNAs and that mitochondrial IMS RNase T2 (RNASET2) selectively degrades the rRNAs. The RNase activity also has a positive effect on nuclear transcription of rRNAs and affects translation within mitochondria and that on the outer surface of mitochondrial outer membrane. These findings establish a mechanism for regulation of cytosolic ribosomes and co-regulation of mitochondrial and cytosolic translation programs by mitochondria.

## Results

### In organello decay of mitochondrion-associated cytosolic rRNAs

**Figure 1.**
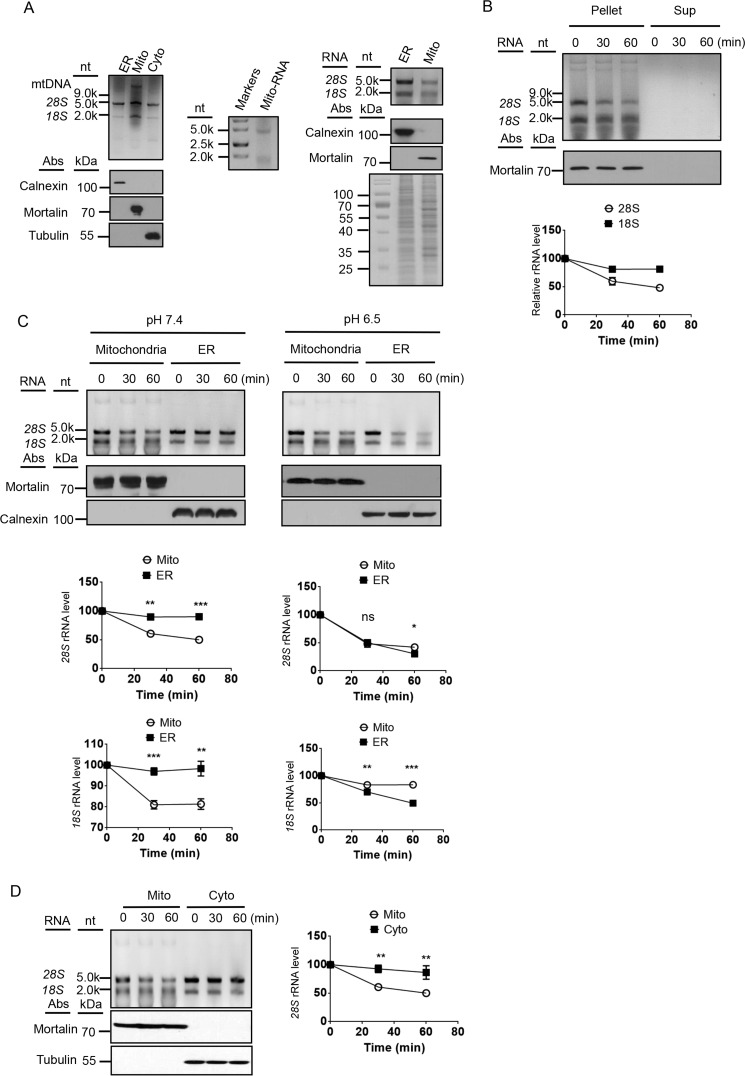
**Degradation of mitochondrion-associated cytosolic rRNAs.** *A*, comparison of total ER, mitochondrial (*Mito*) and cytosolic (*Cyto*) nucleic acids. The *top panel* on the *left* shows the nucleic acids on an EtBr agarose gel. Equal cell volume of mitochondria and ER were loaded. The *bottom panels* on the *left* display the immunoblots of ER, cytosolic, and mitochondrial markers. The *middle panel* shows the total mitochondrial RNAs with RNA markers on a denaturing gel. The *top panel* on the *right* displays nucleic acids in equal protein volume of ER and mitochondria. The *bottom panel* on the *right* displays the Coomassie staining of ER and mitochondrial lysates. *nt*, nucleotides. *B*, *in organello* decay of mitochondrion-associated cytosolic rRNAs. The *top panel* shows mtDNA and mitochondrion-associated cytosolic rRNAs in the mitochondrial pellets or the incubation buffer (*Sup*) at three time points. The *middle panel* shows the immunoblot of the samples. The *bottom panel* displays the quantification of the rRNAs (*n* = 3). *C*, *in organello* decay of mitochondrion-associated cytosolic rRNAs and ER-associated rRNAs at pH 7.4 and pH 6.5. *D*, comparison of the decay of mitochondrion-associated cytosolic rRNAs (*Mito*) and rRNAs in the cytosol (*Cyto*). Statistical comparisons are performed using unpaired *t* tests (*n* = 3 if not specified). *, *p* < 0.05; **, *p* < 0.01; ***, *p* < 0.001; ****, *p* < 0.0001. The data are presented as means ± S.D.

During our previous attempt to understand mtRNA degradation, we examined the total nucleic acids isolated from mitochondria. A few bands, including a mitochondrial DNA band, were observed after the samples were run on an agarose gel with ethidium bromide (EtBr) (Fig. 1*A*). Originally, we thought the two major RNA bands were 16S and 12S mitochondrial rRNAs. However, by comparing them to molecular markers and RNAs isolated from cytosol and purified ER, we realized that the two major RNA bands are 28S and 18S cytosolic rRNAs (Fig. 1*A*). It has been reported that a fraction of cytosolic ribosomes bind tightly to mitochondrial outer membrane and that some cytosolic mRNAs are also associated with mitochondria (19, 20, 23). We estimated that in equal cell volume the amount of rRNAs on mitochondria is approximately the same as that on ER and that in equal protein volume the amount of rRNAs on mitochondria is ∼30% of that on ER in HEK cells (Fig. 1*A*). Based on the number and the Western controls, it seems unlikely that the cytosolic rRNAs on purified mitochondria are from ER contamination.

Next we examined how strong the binding between the cytosolic rRNAs and the mitochondria is. After 1 h of incubation in an isotonic buffer, very little dissociation of the rRNAs from mitochondrial outer membrane occurred (Fig. 1*B*). However, a decrease of the amount of the RNAs was observed. The rate of 28S rRNA decay appeared to be faster than that of 18S rRNA (Fig. 1*B*). In addition, the patterns of decay on isolated mitochondria were vastly different from those on ER. At pH 7.4, very little degradation occurred on ER after 1 h of incubation, whereas ∼50% of the 28S rRNA was degraded in the same period of time on mitochondria (Fig. 1*C*). At pH 6.5, however, degradation rate on ER increased dramatically but only slightly on mitochondria (Fig. 1*C*). The degradation patterns on mitochondria were also very different from those of the soluble cytosolic rRNAs with the rate of 28S rRNA on mitochondria much faster than that of the cytosolic 28S rRNA (Fig. 1*D*). Taken together, these results demonstrate that mitochondria contain a distinct pool of cytosolic rRNAs that are degraded by a different mechanism from that of the ER rRNAs or the soluble rRNAs.

### Cytosolic rRNAs on isolated mitochondria are not degraded outside of mitochondria

**Figure 2.**
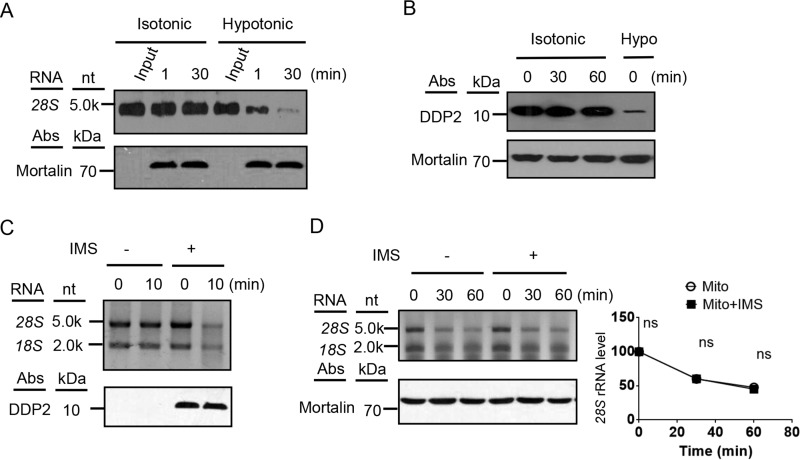
**Mitochondrion-associated cytosolic rRNAs are not degraded by a cytosolic nuclease.** *A*, no RNase activity resides at the outer surface of mitochondrial outer membrane. Isolated mitochondria were resuspended in MitoPrep buffer (*Isotonic*) or hypotonic buffer that ruptures the mitochondrial outer membrane. Biotinylated 28S *r*RNA was added to the mixture and incubated at 37 °C for 1 or 30 min before the reaction was terminated. The *bottom panel* shows the mortalin immunoblot of the samples. *nt*, nucleotides. *B*, immunoblotting of a mitochondrial IMS protein DDP2 and matrix protein mortalin in the *in organello* degradation mitochondrial samples at 0, 30, and 60 min and the 0-min sample in the hypotonic buffer (*Hypo*). *C*, purified mitochondrial IMS fraction was tested for nuclease activity with purified cytosolic RNAs as substrates. The *bottom panel* shows the immunoblot of mitochondrial IMS protein DDP2. *D*, *in organello* decay of mitochondrion-associated cytosolic rRNAs with or without the addition of purified mitochondrial IMS fraction. Statistical comparisons are performed using unpaired *t* tests (*n* = 3 if not specified). *, *p* < 0.05; **, *p* < 0.01; ***, *p* < 0.001; ****, *p* < 0.0001. The data are presented as means ± S.D.

To understand how cytosolic rRNAs are degraded on isolated mitochondria, we first examined whether there is a RNase activity on the outside of mitochondrial outer membrane. *In vitro* synthesized 28S rRNA fragment was incubated with isolated mitochondria in an isotonic buffer or a hypotonic buffer that ruptures the mitochondrial outer membrane. In the isotonic buffer, no degradation of the added 28S rRNA occurred, but in the hypotonic buffer, the added 28S rRNA was quickly degraded, indicating that there is no RNase activity on the outer surface of the mitochondrial outer membrane (Fig. 2*A*). Instead, a strong RNase activity resides in the mitochondrial IMS, consistent with the previous results (9). The integrity of the outer membrane during 1 h of incubation in the isotonic buffer was also examined. No leakage of the soluble mitochondrial IMS protein DDP2 was observed at the end of the incubation (Fig. 2*B*). By contrast, majority of DDP2 was leaked out of mitochondria in the hypotonic buffer that ruptures the outer membrane but leaves the inner membrane intact (Fig. 2*B*). We still could not rule out the possibility that the small amount of IMS leaked during incubation is responsible for the decay of mitochondrion-associated cytosolic rRNAs, so we isolated the mitochondrial IMS and added it into the *in organello* degradation mixture. Purified IMS readily degraded TRIzol purified rRNAs but had no significant effect on the decay of the mitochondrion-associated cytosolic rRNAs (Fig. 2, *C* and *D*). These results have three implications: first, the cytosolic rRNAs on the mitochondrial outer membrane are most likely in the ribosomes because they seem to be resistant to the exogenously added IMS; second, an active extraction of the rRNAs from the ribosomes should be involved in the *in organello* decay; and third, rRNAs on the outer surface of the mitochondrial outer membrane are degraded within mitochondria.

### Characterization of in organello rRNA degradation of mitochondrion-associated cytosolic rRNAs

**Figure 3.**
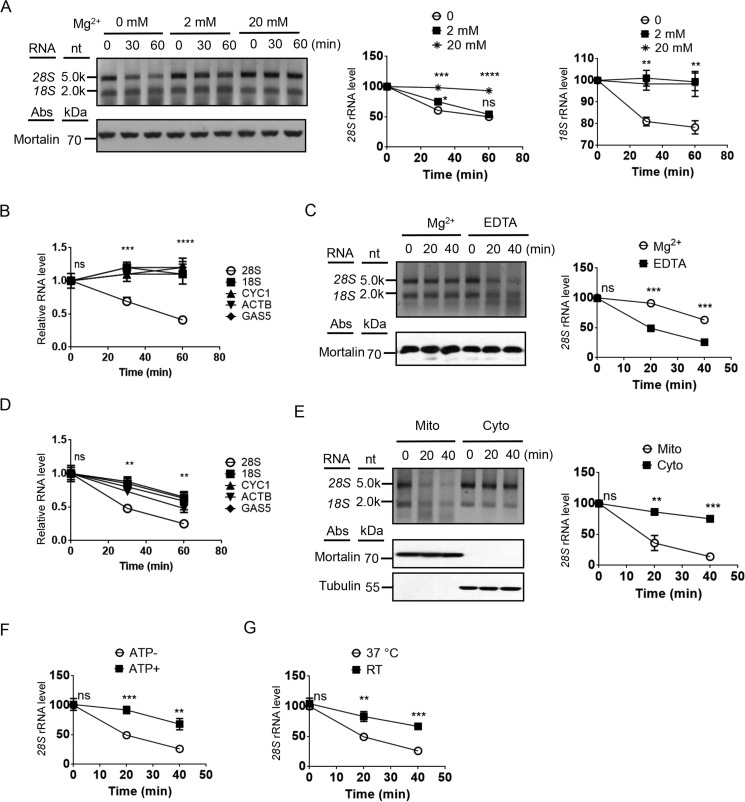
***In organello* degradation of mitochondrion-associated cytosolic rRNAs are sensitive to Mg^2+^, EDTA, ATP, and temperature.** *A*, *in organello* degradation of mitochondrion-associated cytosolic rRNAs with or without Mg^2+^. The graphs on the *right* display the quantification of 18S and 28S rRNAs (*n* = 3). *nt*, nucleotides. *B*, *in organello* degradation of mitochondrion-associated RNAs with 2 mm Mg^2+^. RNAs were isolated from the degradation samples, and qRT-PCR was performed. *C*, *in organello* degradation of mitochondrion-associated 28S and 18S rRNAs with 2 mm Mg^2+^ or 2 mm EDTA. *D*, *in organello* degradation of mitochondrion-associated RNAs with EDTA. *E*, comparison of the decay of mitochondrion-associated cytosolic rRNAs (*Mito*) and rRNAs in the cytosol (*Cyto*) with EDTA. *F*, *in organello* degradation of mitochondrion-associated 28S with EDTA and with or without 8 mm ATP. *G*, *in organello* degradation of mitochondrion-associated 28S with EDTA at 37 °C or room temperature. Statistical comparisons are performed using unpaired *t* tests (*n* = 3 if not specified); *, *p* < 0.05; **, *p* < 0.01; ***, *p* < 0.001; ****, *p* < 0.0001. The data are presented as means ± S.D.

Because the mitochondrion-associated cytosolic rRNAs are most likely in the ribosomes, we investigated whether stabilizing or destabilizing the ribosomes has any effect on the degradation of the rRNAs. Mg^2+^ is essential for ribosome stability and has been shown to be involved in regulation of RNase activities (9, 27). A small amount of Mg^2+^ (2 mm) appeared to have only minor effect on 28S rRNA degradation but completely inhibited the degradation of 18S rRNA, whereas higher concentration of Mg^2+^ (20 mm) blocked both 18S and 28S rRNA degradation (Fig. 3*A*). qRT-PCR of the RNA samples isolated from mitochondria at different time points also show that in addition to 18S rRNA, other mitochondrion-associated RNAs were not degraded in the presence of 2 mm Mg^2+^ and that only 28S was selectively degraded under the condition (Fig. 3*B*). Adding metal ion chelator EDTA to the degradation buffer led to a faster degradation of 28S rRNA and other mitochondrion-associated RNAs (Fig. 3, *C* and *D*). In comparison, the effect of EDTA on rRNAs in isolated cytosol was much milder, which is another indication that the degradation of mitochondrion-associated RNAs is not due to contamination of a cytosolic RNase activity (Fig. 3*E*). The degradation of the mitochondrion-associated 28S rRNA was also inhibited by high ATP concentration and lower temperature, two conditions that have been shown to negatively affect certain RNase activities (9) (Fig. 3, *F* and *G*).

### Mitochondrial IMS RNase RNASET2 is involved in degradation of mitochondrion-associated cytosolic RNAs

**Figure 4.**
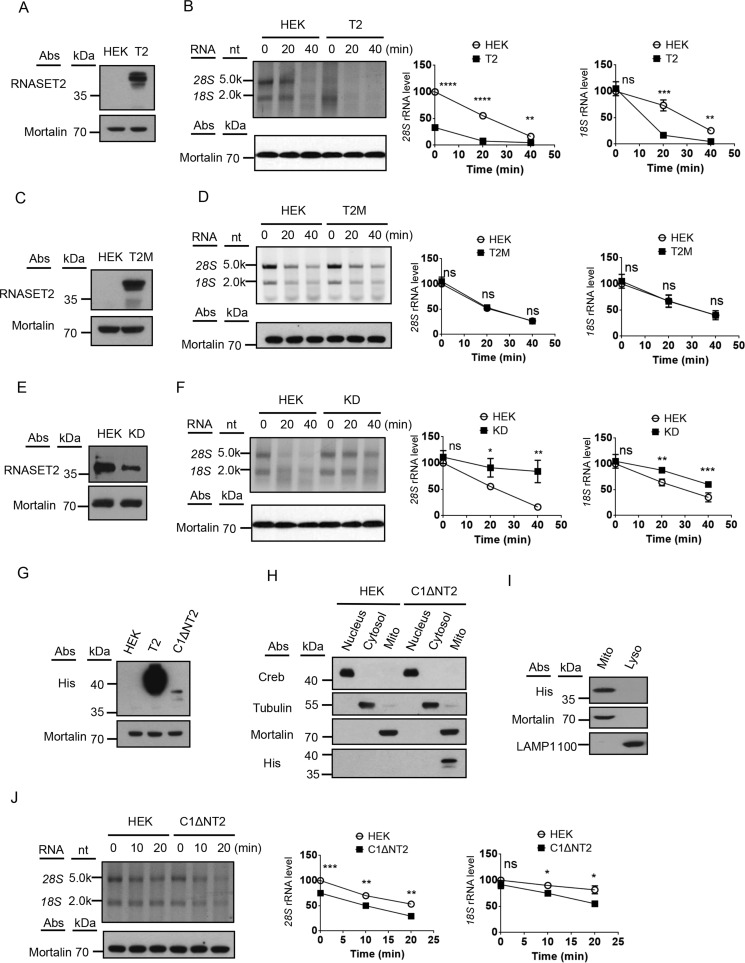
**RNASET2 in the mitochondria is involved in degradation of cytosolic rRNAs on the mitochondrial outer membrane.** *A*, immunoblots of HEK and RNASET2-overexpressing (*T2*) cell lysates. Mortalin was used as a loading control. *B*, *in organello* degradation of mitochondrion-associated 28S and 18S rRNAs on isolated control mitochondria (*HEK*) and mitochondria overexpressing RNASET2 (*T2*) with EDTA. The grafts show quantification of 28S and 18S rRNAs. *nt*, nucleotides. *C*, immunoblots of the lysates of HEK cells and cells overexpressing the catalytically inactive RNASET2(H65Y, H118Y) mutant (*T2M*). Mortalin was used as a loading control. *D*, *in organello* degradation of mitochondrion-associated 28S and 18S rRNAs on isolated control mitochondria (*HEK*) and mitochondria overexpressing RNASET2(H65Y, H118Y) with EDTA. The grafts show quantification of 28S and 18S rRNAs. *E*, immunoblots of HEK or RNASET2 knockdown (*KD*) cell lysates. Mortalin was used as a loading control. *F*, *in organello* degradation of mitochondrion-associated 28S and 18S rRNAs on isolated control mitochondria (*HEK*) and RNASET2 knockdown mitochondria (*KD*) with EDTA. *G*, immunoblots of lysates of HEK cells and cells overexpressing RNASET2 (*T2*) or C1-ΔNRNASET2 (*C1*Δ*NT2*). Mortalin was used as a loading control. *H*, immunoblots of different cellular fractions from HEK cells and cells overexpressing C1-ΔNRNASET2 (*C1*Δ*NT2*): the nucleus, the cytosol and mitochondria (*Mito*). Creb, β-tubulin, and mortalin were used as markers for the nucleus, the cytosol, and mitochondria, respectively. *I*, immunoblots of mitochondria (*Mito*) and lysosomes (*Lyso*) isolated from cells expressing C1-ΔN-RNASET2 (*C1*Δ*NT2*). LAMP1 was used as a lysosomal marker, and mortalin was used as a mitochondrial marker. *J*, *in organello* degradation of mitochondrion-associated 28S and 18S rRNAs on isolated control mitochondria (*HEK*) and mitochondria overexpressing C1-ΔN-RNASET2 (*C1*Δ*NT2*) with EDTA. Statistical comparisons are performed using unpaired *t* tests (*n* = 3 if not specified). *, *p* < 0.05; **, *p* < 0.01; ***, *p* < 0.001; ****, *p* < 0.0001. The data are presented as means ± S.D.

It has been shown previously that the IMS RNASET2 is directly involved in degradation of mtRNA and processing of noncoding RNA imported from the cytosol into mitochondria (9, 28). The RNase RNASET2 is sensitive to multiple cellular conditions, such as pH, Mg^2+^, temperature, and ATP (9). The characteristics of *in organello* degradation of mitochondrion-associated cytosolic RNAs show a striking resemblance to those of mtRNAs degradation and the RNase RNASET2 (9). To examine whether mitochondrion-localized RNASET2 is involved in degradation of the mitochondrion-associated cytosolic rRNAs, cell lines were constructed with RNASET2 overexpressed or knocked down. Mitochondria were isolated and *in organello* degradation was performed. On RNASET2-overexpressing mitochondria, the 28S rRNA level was much lower, making it difficult to compare the degradation rate directly, but the degradation of 18S rRNA was clearly faster on the RNASET2-overexpressing mitochondria (Fig. 4, *A* and *B*). Mutations (H65Y and H118Y) that result in a catalytically inactive form of RNASET2 (29) abolished the effect of RNASET2 overexpression on degradation of mitochondrion-associated cytosolic rRNAs (Fig. 4, *C* and *D*). Knockdown of RNASET2 led to slower degradation of the mitochondrion-associated 18S and 28S rRNAs (Fig. 4, *E* and *F*). Because RNASET2 is a protein with multiple subcellular localizations, the effects of overexpression and knockdown could be indirect (9, 30). To rule out the possibility, we fused RNASET2 without the N-terminal 24 amino acids to the targeting sequence of cytochrome C1 that targeted the protein to the IMS and expressed the fusion protein (C1ΔNT2) in HEK cells (Fig. 4, *G–I*). Subcellular fractionation showed that C1ΔNT2 is localized in mitochondria (Fig. 4, *H* and *I*). Expression of the C1ΔNT2 had a similar but milder effect on degradation of mitochondrion-associated cytosolic rRNAs as overexpression of RNASET2. The milder effect is likely due to the much lower expression level of the fusion protein compared with that of the WT RNASET2 (Fig. 4*G*).

### RNASET2 regulates the amount of mitochondrion-associated cytosolic rRNAs in vivo and has a positive effect on nuclear transcription

**Figure 5.**
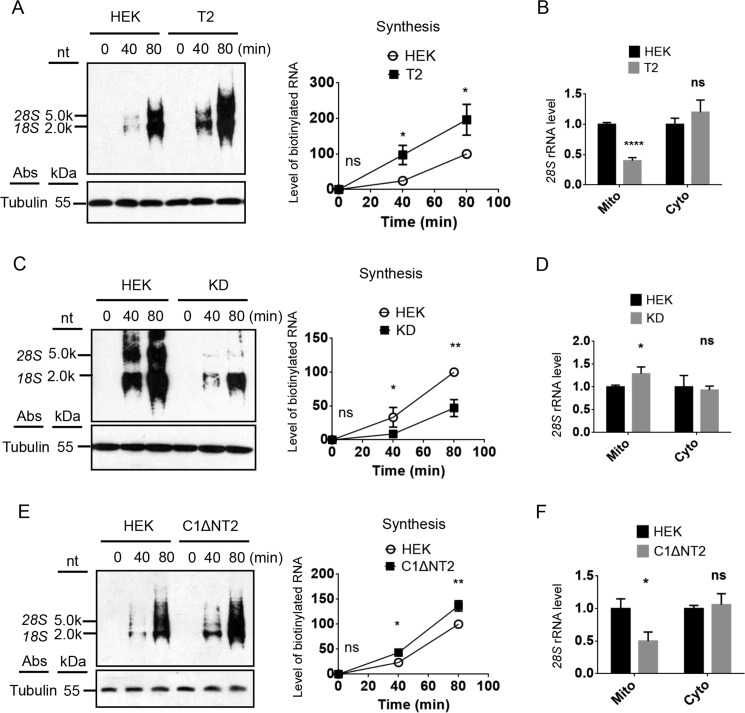
**Mitochondrial RNASET2 regulates cytosolic rRNA levels and nuclear transcription *in vivo*.** *A*, *in vivo* RNA synthesis in HEK and RNASET2-overexpressing cells (*T2*). The *lower panel* on the *left* is an immunoblot of tubulin. The *right panel* shows the quantification of biotinylated RNAs. *nt*, nucleotides. *B*, qRT-PCR results of mitochondrial and cytosolic 28S rRNA in HEK, and RNASET2-overexpressing (*T2*) cells. *C*, *in vivo* RNA synthesis in HEK and RNASET2 knockdown cells (*KD*). *D*, qRT-PCR results of mitochondrial and cytosolic *28S* rRNA in HEK, and RNASET2 knockdown (*KD*) cells. *E*, *in vivo* RNA synthesis in HEK cells and cells expressing C1-ΔN-RNASET2 (*C1*Δ*NT2*). *F*, qRT-PCR results of mitochondrial and cytosolic 28S rRNA in HEK and C1-ΔN-RNASET2 expressing (*C1*Δ*NT2*) cells. Statistical comparisons are performed using unpaired *t* tests (*n* = 3 if not specified). *, *p* < 0.05; **, *p* < 0.01; ***, *p* < 0.001; ****, *p* < 0.0001. The data are presented as means ± S.D.

To examine whether the *in organello* findings are relevant *in vivo*, the levels of nuclear transcription, cytosolic rRNA, and mitochondrion-associated cytosolic rRNAs were examined. *In vivo*, the level of an RNA is the result of a fine balance among synthesis, transport, and degradation. To examine whether manipulation of RNASET2 level has an effect on nuclear rRNA transcription, *in vivo* RNA synthesis was performed using live cells and biotin RNA labeling mix (Roche). Two major biotin-labeled rRNA bands were observed. Remarkably, RNASET2 overexpression led to a 2-fold up-regulation of nuclear rRNA synthesis (Fig. 5*A*). The level of mitochondrial 28S rRNA in the RNASET2-overexpressing cells was approximately one-third of that in the control HEK cells, even though the cytosolic levels were approximately the same (Fig. 5*B*). A faster synthesis rate and similar cytosolic level combined with a lower mitochondrial 28S level *in vivo* suggests a faster degradation, consistent with the *in organello* degradation results. Knockdown of RNASET2 also had a dramatic effect on nuclear transcription, with the transcription rate dropping to approximately one-third of that in the control cells (Fig. 5*C*). Only a small increase of mitochondrial 28S rRNA level was observed, mostly likely because of the strong feedback down-regulation of nuclear RNA synthesis (Fig. 5*D*). However, a slightly higher mitochondrial 28S rRNA level combined with a significantly lower synthesis rate also suggests a slower degradation *in vivo* in the RNASET2 knockdown cells. Expression of the mitochondrion-targeted fusion protein C1ΔNT2 had a similar effect on nuclear transcription and on the level of the mitochondrion-associated cytosolic rRNAs as RNASET2 overexpression (Fig. 5, *E* and *F*). Taken together, these results demonstrate that mitochondrial RNASET2 regulates not only the mitochondrion-associated cytosolic ribosomes but also nuclear transcription possibly through a compensatory feedback.

### Mitochondrion-associated cytosolic rRNAs enter IMS through the mitochondrial RNA import pathway

**Figure 6.**
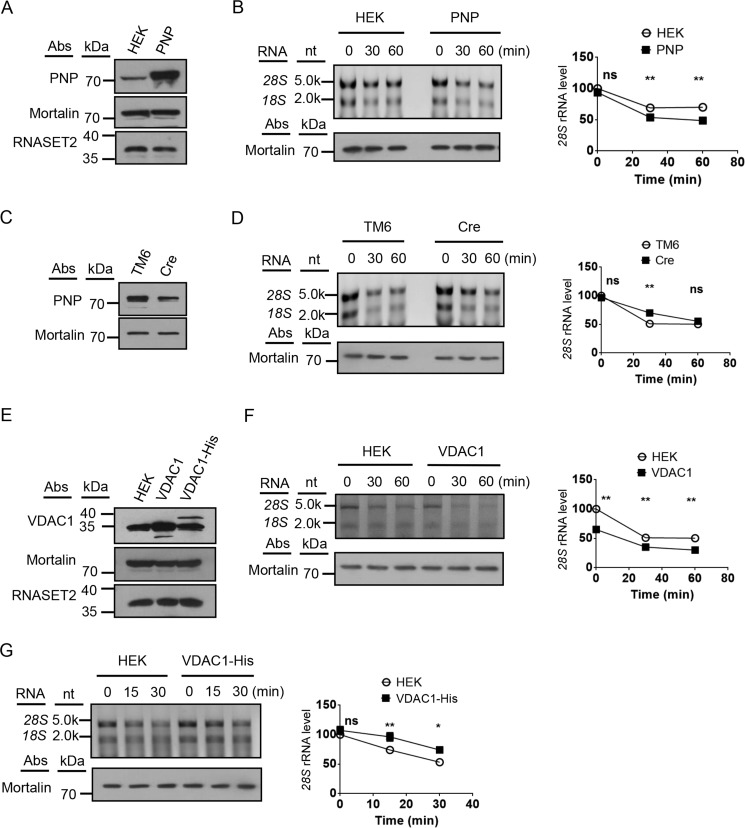
**Cytosolic rRNAs are transported to the IMS RNA degradation machinery through the mitochondrial RNA import pathway.** *A*, immunoblots of HEK and PNPASE-overexpressing (*PNP*) cell lysates. Mortalin was used as a loading control. *B*, *in organello* degradation of mitochondrion-associated 28S and 18S rRNAs on isolated control mitochondria (*HEK*) and mitochondria overexpressing PNPASE (*PNP*) with 2 mm ATP. The grafts show quantification of 28S rRNAs. *C*, immunoblots of TM6 and PNPASE knockdown TM6 (*Cre*) cell lysates. Mortalin was used as a loading control. *D*, *in organello* degradation of mitochondrion-associated 28S and 18S rRNAs on isolated control mitochondria (*TM6*) and PNPASE knockdown mitochondria (*Cre*). The grafts show quantification of 28S rRNAs. *E*, immunoblots of the lysates of HEK cells harboring an empty vector (*HEK*), overexpressing VDAC1, or expressing VDAC1 with a His Tag (VDAC1-His). Mortalin was used as a loading control. *F*, *in organello* degradation of mitochondrion-associated 28S and 18S rRNAs on isolated control mitochondria (*HEK*) and mitochondria overexpressing VDAC1 (*VDAC1*). The grafts show quantification of 28S rRNAs. *G*, *in organello* degradation of mitochondrion-associated 28S and 18S rRNAs on isolated control mitochondria (*HEK*) and mitochondria overexpressing VDAC1 with a His tag (*VDAC1-His*). The grafts show quantification of 28S rRNAs. Statistical comparisons are performed using unpaired *t* tests (*n* = 3 if not specified). *, *p* < 0.05; **, *p* < 0.01; ***, *p* < 0.001; ****, *p* < 0.0001. The data are presented as means ± S.D.

To understand substrate delivery in degradation of mitochondrion-associated cytosolic rRNAs, the existing mitochondrial RNA import pathway was examined. Mammalian PNPASE has been shown to be a major component of the pathway (2). Overexpression of PNPASE in HEK cells led to an acceleration of cytosolic rRNA degradation in the isolated mitochondria without affecting RNASET2 protein level, whereas knockdown of the protein in an immortalized mouse cell line TM6 decreased the degradation rate (Fig. 6, *A–D*). Another known component of mitochondrial RNA import pathway is voltage-dependent anion channel (VDAC) in the mitochondrial outer membrane (31). Overexpression of VDAC1 in HEK cells led to a decrease of the cytosolic rRNA level on the mitochondrial outer membrane without affecting RNASET2 protein level, suggesting a faster degradation rate (Fig. 6, *E* and *F*). When a negatively charged His tag was added to the C terminus of VDAC1, the fusion protein inhibited the degradation of cytosolic rRNA in the mitochondria, suggesting that electrostatic interaction between RNA and VDAC plays an important role in cytosolic rRNA import and degradation (Fig. 6, *E* and *G*).

### The mitochondrial IMS RNASET2 regulates translation in and out of mitochondria

**Figure 7.**
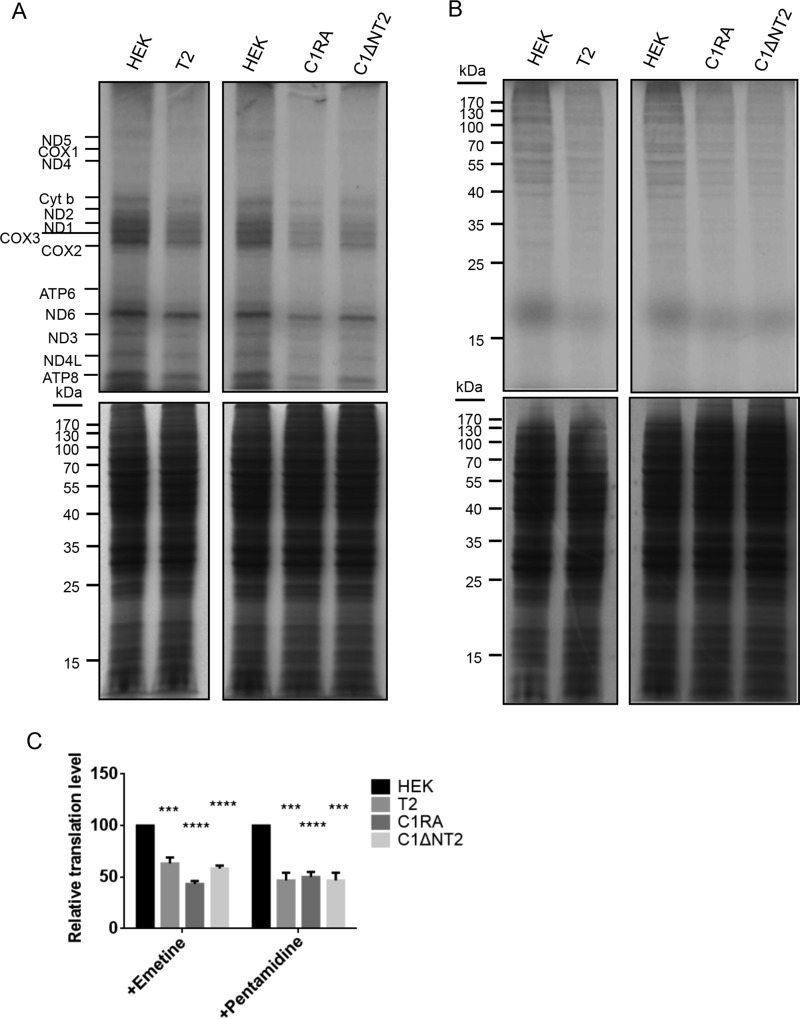
**Mitochondrial IMS degradation machinery regulates translation within mitochondria and translation on the mitochondrial outer membrane.** *A*, *in organello* translation of mitochondria isolated from HEK cells harboring an empty vector, overexpressing RNASET2, expressing C1-RNase A (*C1RA*), or expressing C1-ΔNRNASET2 (*C1*Δ*NT2*) with emetine. *Upper panels*, [^35^S]methionine and cysteine labeling of newly synthesized proteins; *lower panels*, Coomassie staining of SDS gels with mitochondrial proteins. *B*, *in organello* translation of mitochondria with pentamidine. *C*, quantification of the relative translation levels in *A* and *B*. Statistical comparisons are performed using unpaired *t* tests (*n* = 3 if not specified). *, *p* < 0.05; **, *p* < 0.01; ***, *p* < 0.001; ****, *p* < 0.0001. The data are presented as means ± S.D.

Mitochondrial proteins are encoded in both mitochondrial genome and nuclear genome. The expressions of these two pools of mitochondrial genes are co-regulated and synchronized (14). It has been shown that most inner membrane proteins are translated on the outer membrane (19). To understand the biological function of cytosolic rRNA degradation by mitochondrial RNASET2, translation programs within mitochondria and on the mitochondrial outer membrane were examined using ^35^S-labeled methionine and cysteine in the presence of emetine or pentamidine, which enables observation of mitochondrial translation or cytosolic translation separately. Overexpression of RNASET2 not only inhibits mitochondrial translation but also inhibits cytosolic translation on the outer membrane of isolated mitochondria (Fig. 7, *A–C*). Expression of RNase A or RNASET2 that is targeted specifically to mitochondrial IMS with cytochrome *c*_1_–targeting signal (C1RA or C1ΔNT2) also had a similar effect on both the mitochondrial translation and cytosolic translation programs (Fig. 7, *A–C*), suggesting co-regulation of the two translation programs by the IMS RNASET2.

## Discussion

Mitochondrial complexes are assembled from subunits encoded in the nucleus and the mitochondria. How to coordinate the synthesis, transport, and assembly of the subunits with dual origins has been one of the central questions in biology sciences. Much progress has been made in recent years. Mitochondrial and cytosolic translation programs have been shown to be synchronized in *Saccharomyces cerevisiae* (14). Localized translation at mitochondrial outer membrane is also coupled with protein import and complex assembly (19), and mitochondrial ribosomes display translational plasticity in response to the influx of nucleus-encoded OXPHOS subunits (24, 25). However, it was unknown whether the cytosolic translation could be regulated by mitochondria. Here, we show that rRNAs on the mitochondrial outer membrane are degraded by the RNASET2 in the mitochondrial IMS in mammals. Interestingly, the degradation activity also shows a positive correlation with nuclear transcription, suggesting a compensatory feedback. Both mitochondrial translation and cytosolic translation on the mitochondrial outer membrane are also regulated by mitochondrial RNASET2. These findings provide a mechanism for co-regulating of gene expression programs in and out of mitochondria in mammals. Interestingly, the cytosolic rRNA degradation pathway seems to resemble a newly discovered mitochondrial proteolytic pathway that degrades cytosolic protein aggregates (32).

The IMS RNASET2 that is responsible for degradation of mitochondrion-associated cytosolic rRNAs is also directly involved in degradation of mtRNAs and is sensitive to multiple cellular conditions, such as pH, Mg^2+^, temperature, and ATP (9). It has a positive feedback not only on nuclear transcription but also on mitochondrial transcription. That the synthesis and degradation of both mitochondrial rRNAs and the cytosolic rRNAs are regulated by such an activity suggests coordination of the gene expression programs inside and outside of mitochondria in mammals. Localized translation is prevalent in cells and has great biological significance (15–17). It is not surprising that a similar strategy is adopted by RNA degradation. Interestingly, the IMS RNASET2 degrades mitochondrion-associated 28S rRNA faster than most other cytosolic RNAs *in vivo*. A quick change of rRNA normally correlates with cellular events that require fast changes in protein synthesis, such as cell proliferation and differentiation (33, 34). These events usually are also accompanied by changes of energy requirement and mitochondrial activity (35). Mitochondrial localization of such a RNase would efficiently coordinate these events with mitochondrial biosynthesis and energy output. It also provides a mechanism on how mitochondria biosynthesis is regulated in response to different cellular conditions.

The role of RNASET2 in rRNA decay appears to be conserved. One of the RNASET2 enzymes RNS2 in *Arabidopsis thaliana* is localized to ER and vacuoles (36). Unlike most protozoan RNASET2 enzymes that have an acidic pH preference and reside in lysosomes, RNS2 has a preference for neutral pH that enables it to function in ER (36). This result is consistent with mammalian RNASET2 activity that also has a more neutral pH preference (9). In *Arabidopsis* mutants lacking RNS2 activity, rRNAs have longer half-lives, suggesting that RNS2 is required for rRNA degradation in plants (36). A similar function of RNASET2 was also reported in zebrafish (37). In addition, it has been reported that several RNASET2 enzymes in the ciliate *Tetrahymena* appear to be involved in tRNAs and rRNA turnover (38). Here, we show that in mammals, ER also appears to have an rRNA degradation machinery, because rRNAs on isolated ER show a decay pattern that is different from that of the cytosolic rRNAs on mitochondria. Whether RNASET2 is also involved in ER rRNA degradation in mammals remains to be examined.

To be degraded in the mitochondrial IMS, cytosolic RNAs have to be transported across the mitochondrial outer membrane. Import of cytosolic RNAs into mitochondria is a common process across species, and the known substrates include 5S rRNA, tRNAs, and other noncoding RNAs (2, 3, 39–43). We have shown that cytosolic 18S and 28S rRNAs share the import pathway with these RNAs, but what determines certain RNA to be degraded or not remains to be elucidated. Different factors including sequences, secondary structures, tertiary structures, and binding partners could play a role in which RNAs are destined for degradation.

We show that the IMS RNA degradation activity is co-regulated with the nuclear transcriptional activity to maintain relatively stable cytosolic RNA levels. However, virtually nothing is known about the mechanism of this co-regulation. A specific signaling pathway is clearly what is required to bridge the cross-talk between mitochondria and the nucleus. More studies are needed to identify and characterize the pathway.

## Experimental procedures

### Cell lines and culture

Cells were cultured in Dulbecco's modified Eagle's medium supplemented with 10% fetal bovine serum. To generate stable cell lines, the cells were co-transfected with the constructs of interest plus *VSVg* and *Hit60* packaging vectors using TurboFect (Thermo). Harvested retroviruses were used to infect HEK293 cells, followed by selection with 1 μg/ml puromycin. RNASET2 knockdown was achieved using shRNA-expressing constructs (Sigma–Aldrich).

### Plasmids

Plasmids used include *PQCXIP-RNASET2-HAHisPC*, *PQCXIP-C1-*Δ*N-RNASET2-HAHisPC*, *PQCXIP-RNASET2* (*H65Y*, *H118Y*), *PQCXIP-C1-RNASEA*, *PQCXIP-VDAC1*, and *PQCXIP-VDAC1-HisFlag. PQCXIP-C1-RNASEA* was constructed in the same way as *PQCXIP-C1-*Δ*N-RNASET2-HAHisPC*, with *RNASEA* PCR amplified from HEK293 cDNA with primers 5′-TCCACCGGTATGGCTCTGGAGAAGTCT-3′ and 5′-CGCGGATCCGGTAGAGTCCTCCACAG-3′. To construct *PQCXIP-VDAC1* and *PQCXIP-VDAC1-HisFlag*, *VDAC1* was PCR-amplified from HEK293 cDNA library using the primers 5′-ATATAGCGGCCGCATGGCTGTGCCACCCAC-3′ and 5′-AGCGCTCGAGTGCTTGAAATTCCAGTCCTAGACC-3′ and inserted into *PQCXIP* and *PQCXIPHisFlag* with NotI and XhoI. To construct *PQCXIP-RNASET2* (*H65Y*, *H118Y*) mutant, *RNASET2 H65Y* and *H118Y* site-directed mutagenesis were performed using the following primers: H65Y forward, 5′-GGACAATATATGGACTATGGCCCGAT-3′; H65Y reverse, 5′-CCATATATTGTCCAGTAATCCGGAGGG-3′; H118Y forward, 5′-GGAAAAGTATGGGACCTGCGC-3′; and H118Y reverse, 5′-CCCATACTTTTCCCACTCATGC-3′. To construct *PQCXIP-C1-*Δ*N-RNASET2-HAHisPC*, Δ*N-RNASET2-HA* was PCR-amplified from *PQCXIP-RNASET2-HAHisPC* with primers 5′-TCCACCGGTATGGACAAGCGCCTGCGT-3′ and 5′-AAAGGATCCGGCGTAATCTGGAACATCGTATGGGTAACTTGTTCTATGCTTGGTCTTTT-3′ and inserted into *PQCXIP-C1-HisPC* (9) with AgeI and BamHI.

### Isolation of crude mammalian mitochondria, ER, the cytosol, and the nucleus

The cells were washed once with PBS buffer, resuspended in ice-cold MitoPrep buffer (0.225 m mannitol, 0.075 m sucrose, and 20 mm HEPES, pH 7.4) and processed in a Dounce homogenizer 30 times on ice in a glass Teflon homogenizer. Nuclei and unbroken cells were pelleted at 700 × *g* for 5 min, and the homogenization was repeated once. Supernatants from both times were centrifuged again at 700 × *g*. Crude mitochondria were pelleted from second-round supernatants at 11 kg for 5 min, washed once with MitoPrep buffer, and resuspended in MitoPrep buffer of desired volume for further use. Postmitochondrial supernatant was spun at 100 kg for 10 min, and the supernatant was collected as cytosol. For ER isolation on gradient, crude ER was pelleted at 21 kg for 10 min. For nuclear fraction isolation, the pelleted nuclei and unbroken cells were lysed in buffer A (10 mm HEPES, pH 7.9, 10 mm KCl, 1.5 mm MgCl_2_, 0.5% Nonidet P-40) on ice for 20 min. The nuclei were spun down at 1.5 kg for 4 min and washed once with buffer A.

### OptiPrep gradient centrifugation

Crude mitochondria or ER were resuspended in 19% OptiPrep (Sigma, diluted with MitoPrep buffer), layered in 5-ml OptiPrep gradients (8, 12, 16, 19, 22.5, and 27%), and centrifuged at 150 kg for 4 h at 4 °C in a Beckman SW55-Ti rotor. 9 fractions were collected from the top, diluted with 1 ml of MitoPrep buffer, and pelleted at 21 kg for 10 min.

### Submitochondrial fractionation

Hypotonic treatment was performed by incubating mitochondria for 20 min on ice by diluting MitoPrep buffer with 10 volumes of 20 mm HEPES, pH 7.4, with one gentle vortexing at 10 min. IMS was isolated by centrifugation of the hypotonic solution at 15 kg for 10 min.

### Western blotting

The cells were washed twice in 1× PBS, pH 7.4, and lysed in buffer A (10 mm HEPES, pH 7.9, 10 mm KCl, 1.5 mm MgCl_2_, and 0.5% Nonidet P-40). Lysates were spun at 21 kg for 5 min to rid of nuclei. Mitochondria and ER were lysed directly in 1× SDS loading buffer. Solubilized mitochondrial fraction samples were prepared by adding 2× or 4× loading buffer depending on the concentration and the buffers used for solubilization. Protein lysates (50 μg) were resolved by SDS-PAGE, transferred to nitrocellulose membranes, and incubated for 1 h with 5% milk TBS-T and overnight with primary antibodies in 5% BSA at 4 °C or for 1–2 h at room temperature. Antibodies included anti-LAMP1 (1:1000) (Sigma–Aldrich, L1418), anti-calnexin (1:2000) (Cell Signaling Technology, 2433S), anti-tubulin (1:10000) (Cell Signaling Technology, 2146), anti-VDAC1 (1:2000) (Abclonal, A0810), anti-His monoclonal (1:1000) (Abclonal, AE003; Abgent, AM1010a), anti-Creb (1:1000) (Ruiying Biological, RLM3428), anti-RNASET2 (1:1000) (Abgent, AP10764a), anti-mortalin (1:10000) (Sigma–Aldrich, G4045), anti-DDP2 (1:1000), and anti-PNPASE (1:5000) (general gifts from Dr. Carla Koehler at UCLA).

### Mitochondrial RNA isolation

100–200 μg of mitochondria were heated at 70 °C in 100 μl of lysis buffer (1% SDS, 10 mm Tris, pH 7.4, and 10 mm EDTA) for 5 min, cooled to room temperature, and treated with 1 μg of proteinase K at 37 °C for 5 min. 400 μl of TRIzol (Invitrogen) was used for each 100 μl of lysate. The RNA pellet was resuspended in 40 μl of 1× DNase buffer with 0.5 μl of DNase (Thermo) and incubated at 37 °C for 20 min with one vortexing and centrifugation at 10 min. EDTA (5 mm from 50 mm stock) was then added, and the sample was heated at 70 °C for 10 min to inactivate the DNase.

### qRT-PCR

cDNA was synthesized using the Superscript first-strand synthesis system (Invitrogen). SYBR green (Thermo) was used for quantitative PCR.

### In vitro transcription

RNAs were synthesized using the MEGAscript SP6 kit (Ambion) and purified with TRIzol reagent (Invitrogen). Biotin RNA labeling mix (Roche) was used to label the synthesized RNAs.

### In vitro degradation assay

*In vitro* degradation assay was performed as previously described (9). All the assays were performed in 20 mm HEPES, pH 7.4, at 37 °C if not otherwise specified. The reaction was stopped by adding equal volume of SDS–urea–EDTA buffer (2× SDS loading buffer with 8 m urea and 15 mm EDTA) for running on SDS-PAGE or DNA–SDS–EDTA buffer (2× DNA loading buffer with 0.5% SDS and 15 mm EDTA) for running on agarose gel and was incubated at 70 °C for 5 min. The samples were cooled to room temperature, and 0.5 μg of proteinase K was added for a 5-min incubation at 37 °C. Biotinylated samples were run on SDS-PAGE, transferred to a nylon membrane, and detected with nucleic acid detection kit (Thermo). rRNA samples were run on agarose gels with EtBr.

### In vivo RNA synthesis

4 × 10^6^ cells were collected, washed with PBS twice; resuspended in 300 μl of MitoPrep buffer containing 4 mm ATP, pH 7.4, 20 mm succinate, 1 mm CaCl_2_, 10 mm MgCl_2_, and 1 μl of biotin RNA labeling mix (Roche); and incubated at 37 °C. For each time point (0, 40, and 80 min), 60 μl of reaction mix was taken out, and cells were pelleted at 800 × *g* for 1 min. The pellets were stored at −80 °C for at least 15 min before next preparation step. For loading, samples were taken out of −80 °C, quickly dissolved in 50 μl SDS–urea–EDTA buffer (SDS loading buffer with 8 m urea and 15 mm EDTA), and incubated at 70 °C for 5 min. RNA was separated from genomic DNA with an Axygen miniprep column. Each sample was divided into two. 0.2 μg of proteinase K was added in one for a 5-min incubation at 37 °C. The set of samples without proteinase K treatment were used for immunoblotting as loading control, and the set with proteinase K treatment was used for biotin detection of the newly synthesized RNA.

### In organello rRNA degradation

200 μg of mitochondria, 50 μg of ER, or 60 μg of cytoplasm was resuspended in 60 μl of MitoPrep buffer with or without 2 mm Mg^2+^ or 2 mm EDTA on ice. 20 μl was taken out and stored at −80 °C as the first time point sample. The remaining samples were shifted to 37 °C. For each time point (20 and 40 min for samples with EDTA and 30 min and 60 min for the other samples), 20 μl of reaction mix was taken out and stored at −80 °C for at least 15 min before next preparation step. For loading, the samples were taken out of −80 °C, quickly dissolved in 20 μl of DNA–SDS–EDTA buffer (2× DNA loading buffer with 0.5% SDS and 15 mm EDTA), and incubated at 70 °C for 5 min. The samples were then cooled to room temperature, and 0.5 μg of proteinase K was added for a 5-min incubation at 37 °C. The samples were run on 1.5% agarose gel with EtBr.

### In organello translation with l-[^35^S]methionine and cysteine

300 μg of mitochondria were incubated in 50 μl of translation buffer (0.225 m mannitol, 0.075 m sucrose, 1 mm MgCl_2_, 100 mm KCl, 0.05 mm EDTA, 10 mm glutamate, 2.5 mm malate, 1 mm ADP, 1 mg/ml BSA, 10 mm Tris, 10 mm K_2_HPO_4_, pH 7.4, and 10 μm amino acid mix) at 37 °C for 5 min. 100 μCi of l-[^35^S]methionine and cysteine was added, and the mixture was incubated at 37 °C for another 30 min. The samples were then resolved by 12% SDS-PAGE and analyzed by autoradiography.

## Author contributions

J. H., P. L., and G. W. conceptualization; J. H., P. L., and G. W. data curation; J. H. formal analysis; J. H., P. L., and G. W. investigation; J. H., P. L., and G. W. methodology; J. H., P. L., and G. W. writing-original draft; G. W. supervision; G. W. funding acquisition; G. W. project administration; G. W. writing-review and editing.

## Footnotes

2 The abbreviations used are:

OXPHOS: oxidative phosphorylation
IMS: intermembrane space
RNASET2: ribonuclease T2
mtRNA: mitochondrial RNA
EtBr: ethidium bromide
qRT-PCR: quantitative RT-PCR
VDAC: voltage-dependent anion channel
ER: endoplasmic reticulum.
